# Efficacy and safety of lipid-lowering therapies in combination with or without statin to reduce the cardiovascular risk: A systematic review of randomised controlled trials

**DOI:** 10.1016/j.athplu.2024.10.001

**Published:** 2024-10-17

**Authors:** Gabriella Iannuzzo, Geetank Kamboj, Parinita Barman, Shirish Dongare, Shantanu Jawla

**Affiliations:** a Department of Clinical Medicine and Surgery, University of Naples Federico II, Naples, Italy; b Skyward Analytics Pvt. Ltd., Gurugram, India; c Daiichi Sankyo Europe GmbH, Munich, Germany

**Keywords:** Bempedoic acid, Alirocumab, Evolocumab, Ezetimibe, Inclisiran, Cardiovascular outcomes, Cardiovascular risk reduction

## Abstract

**Background and aims:**

Cardiovascular diseases (CVD) pose a significant global health burden. Lowering low-density lipoprotein-cholesterol is the primary therapeutic aim for preventing primary and secondary CVD events. While statins are the standard treatments, their limitations, such as side effects and intolerance in certain patient groups, necessitate exploration of alternative lipid-lowering therapies (LLTs). We systematically reviewed randomised controlled trials (RCTs) evaluating cardiovascular outcomes associated with non-statin LLTs (bempedoic acid, alirocumab, evolocumab, ezetimibe, and inclisiran) in adults with CVD or high cardiovascular risk.

**Methods:**

EMBASE, Medline, Cochrane Library, and clinical trial registries were systematically searched for eligible studies, from inception until February 08, 2023. Two reviewers independently screened the studies, with discrepancies resolved by a third reviewer. Data extraction and validation were conducted, and the risk of bias was assessed using the Cochrane Risk-of-Bias tool-2 for RCTs.

**Results:**

The search strategy yielded 2104 citations. Post screening for eligibility, nine unique trials/studies (84 publications) were identified. Among these, one trial each was identified for bempedoic acid and alirocumab, three for evolocumab, and four for ezetimibe. No published literature documenting the cardiovascular outcomes of inclisiran was identified. Only one trial (CLEAR Outcomes) included statin-intolerant patients at baseline. Most studies evaluated a 3-component, 4-component, or 5-component major adverse cardiovascular events composite as an outcome along with individual components. The quality of the included trials was found to be fair-to-good.

**Conclusions:**

The systematic review findings emphasise the significance of considering non-statin LLTs as viable treatment options for individuals with CVD or high cardiovascular risk who cannot tolerate or achieve optimal lipid control with statin therapy alone.

## Highlights

• First comprehensive review to summarise CVOT trials of non-statin LLTs.
• Non-statin LLTs are crucial for patients who can't tolerate or reach lipid goals with statins.
• Bempedoic acid, alirocumab, evolocumab, and ezetimibe have shown cardiovascular risk reduction benefits.
• Inclisiran trials ongoing, with no current cardiovascular outcomes data.
• Personalized treatment strategies are crucial for managing cardiovascular risk.

## Graphical abstract

**Figure:**
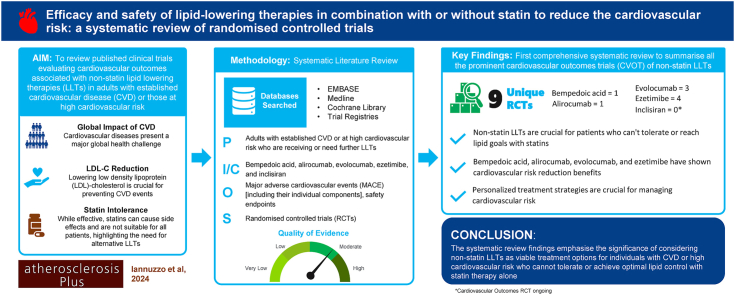

## 1 Introduction

Cardiovascular diseases (CVD) continue to be a major cause of morbidity and mortality globally, which imposes a heavy burden on the healthcare system and society [1,2]. Globally, CVD accounts for roughly 31 per cent of all deaths [3]. Numerous factors contribute to the development of CVD, such as dyslipidaemia, hypertension, obesity, smoking, diabetes mellitus, an unhealthy diet, and physical inactivity [4,5]. Among these factors, dyslipidaemia, particularly elevated low-density lipoprotein cholesterol (LDL-C) levels, is a key contributor to the development of atherosclerosis and subsequent cardiovascular events.

Lowering LDL-C is considered a key therapeutic goal in primary and secondary prevention of CVD [6]. Comprehensive healthcare strategies aim to both prevent the onset of CVD (primary prevention) and reduce the risk of recurrent events (secondary prevention) [7].

The European Society of Cardiology/European Atherosclerosis Society (ESC/EAS) and American College of Cardiology/American Heart Association (ACC/AHA) guidelines suggest aiming for an LDL-C level of under 1.8 mmol/L (<70 mg/dL), or a reduction of over 50 % in LDL-C level from baseline in high-risk patients [8,9]. Statin therapy has been considered the gold standard for managing cholesterol levels and lowering cardiovascular risk [10]. Despite their efficacy, statins are not without limitations. Adverse events (AEs), such as myopathy, hepatotoxicity, and new-onset diabetes mellitus, have been reported with statin use, leading to intolerance and discontinuation of therapy in some patients [11]. Further, many patients are unable to reach their cholesterol goals with statin therapy alone [12]. A meta-analysis revealed a pooled prevalence of statin intolerance at 9.1 % [13]. Statin intolerance was more prevalent in pooled studies encompassing both primary and secondary prevention patients than studies focusing solely on primary or secondary prevention patients [13]. For statin intolerant patients, the cholesterol levels remain uncontrolled, and they remain at increased cardiovascular risk unless alternative lipid-lowering therapies (LLTs) are used. Further, the European SANTORINI real-world study highlights a critical issue in lipid management: the suboptimal outcomes frequently resulting from under prescribing or delays in treatment initiation, which in turn increases the burden of elevated LDL-C levels in patients at high and very high cardiovascular risk [14].

In addition to statin therapy, non-statin LLTs have emerged as valuable adjuncts in lowering cardiovascular risk [8,9,15]. These include novel agents like adenosine triphosphate-citrate lyase (ACL) inhibitors (bempedoic acid), proprotein convertase subtilisin/kexin type 9 (PCSK9) inhibitors (alirocumab and evolocumab), ezetimibe, and small interfering RNA (inclisiran), which target different pathways involved in cholesterol metabolism [16,17]. The meta-analysis from the Cholesterol Treatment Trialists’ Collaboration (CTTC) [18] highlights a direct relationship between the degree of LDL-C reduction and the associated decrease in cardiovascular risk [19]. Clinical trials have demonstrated that the non-statin LLTs mentioned above effectively lower LDL-C in patients with high and very high cardiovascular risk, thereby contributing to cardiovascular risk reduction [[20], [21], [22], [23], [24], [25], [26], [27], [28], [29], [30], [31]]. Guidelines for the management of dyslipidaemias now include the use of non-statin LLTs for statin-intolerant patients and patients at very high-risk atherosclerotic cardiovascular disease (ASCVD) who fail to attain treatment targets despite being on the maximum tolerated dose of a statin [8,9].

Given the evolving landscape of LLTs and the emergence of non-statin agents, there is a need to systematically evaluate the evidence regarding their cardiovascular outcomes. It is essential to conduct a comprehensive analysis of randomised controlled trials (RCTs) investigating the efficacy and safety of non-statin LLTs.

To meet the objective outlined above, we performed a systematic literature review (SLR) to assess published evidence on cardiovascular outcomes associated with non-statin LLTs (bempedoic acid, alirocumab, evolocumab, ezetimibe, and inclisiran) in adults with established CVD or at high cardiovascular risk who are receiving or need further LLTs, including.

• patients on maximally tolerated statins with or without LLTs, placebo; or
• statin-intolerant patients.

## 2 Materials and methods

This SLR followed the principles outlined in the Cochrane Handbook for Systematic Reviews [32]. The methods and results for this SLR are reported in accordance with the Preferred Reporting Items for Systematic Reviews and Meta-Analyses (PRISMA) guidelines [33] *(Supplementary file A)*.

### 2.1 Literature search strategy

The literature searches were conducted in three electronic medical literature databases: EMBASE, Medline, and Cochrane Library, supplemented by searches in two clinical trial registries – ClinicalTrials.gov and the WHO International Clinical Trials Registry Platform (ICTRP). Additionally, a grey literature search was performed using the Google Scholar to identify relevant citations not captured through conventional databases. Moreover, conference proceedings from five major congresses (viz. European Society of Cardiology, Acute Cardiovascular Care, Preventive Cardiology, European Atherosclerosis Society, European Heart Rhythm Association) spanning three years (2020–2022) were manually scrutinised to gather data from citations not yet published as full-text publications. All eligible studies published in English until February 08, 2023, were included.

The search strategy employed key search terms such as ‘cardiovascular disease’, ‘bempedoic acid’, ‘alirocumab’, ‘evolocumab’, ‘ezetimibe’, ‘inclisiran’, and ‘randomised controlled trials’ ‘to retrieve studies of interest *(Supplementary file B)*.

### 2.2 Eligibility criteria

The citations retrieved from databases and grey literature searches were reviewed for eligibility using the PICOS (population, intervention/comparators, outcomes, and study design) framework, ensuring a comprehensive approach to study selection and analysis (Table 1). Citations that failed to meet the eligibility criteria were excluded.

**Table 1:** Inclusion and exclusion criteria (PICOS framework).

	Inclusion criteria	Exclusion criteria
**Population**	Adults with established CVD or at high cardiovascular risk who are receiving or need further LLTs, including: • patients on maximally tolerated statins with or without LLTs, placebo; or • statin-intolerant patients.	• Patients without CVD or who are not at high cardiovascular risk • Paediatric population • Animal/In-vitro studies
**Intervention/Comparators**	Studies focusing on the following interventions alone or in combination with statins or other LLTs – bempedoic acid, alirocumab, evolocumab, ezetimibe, or inclisiran.	Clinical studies that do not investigate one of the interventions of interest in at least one of the arms
**Outcomes**	MACE, including their components and safety endpoints.	Studies that do not report at least one of the outcomes of interest
**Study type**	• Randomised controlled trials. • Systematic reviews were included at the first-level screening only to identify primary studies and were excluded at the second-level (full-text) screening.	• Phase 1 studies • Observational studies • Prognostic studies • Case reports • Case series • Commentaries and letters • Consensus reports • Non-systematic reviews

CVD: Cardiovascular disease; LLTs: Lipid-lowering therapies; MACE: Major adverse cardiovascular events; PICOS: Population, intervention/comparators, outcomes, and study design.

### 2.3 Selection of eligible studies

The titles and abstracts of all citations obtained from databases and grey literature searches were independently screened by two reviewers (GK and PB). Following completion of the first-level screening, full-text screening of potentially relevant or unclear citations was performed by the same reviewers who performed the first-level screening. Any discrepancies were resolved by a third independent reviewer (SD).

### 2.4 Data extraction

Data were extracted in a pre-defined data extraction grid (Microsoft® Excel®) by a single reviewer (GK, PB, or SD), and then independently validated by another reviewer. Extracted data included: trial characteristics (study design, sponsor, sample sizes, location, and follow-up duration), participant details (sex, age, race, weight, body mass index, region, comorbidities, treatment arms with doses, and baseline LDL-C levels), efficacy and safety outcomes data at baseline and various time points. When multiple publications from the same trial were identified, only unique data from the subsequent publications were extracted.

### 2.5 Quality assessment

The quality of each included study was assessed using the Cochrane Risk-of-Bias 2 (RoB 2) tool for RCTs [34]. The RoB 2 tool assesses the risk of bias in five key domains: (a) bias due to the randomisation process, (b) bias due to deviations from the intended interventions, (c) bias resulting from missing outcome data, (d) bias in the measurement of outcomes, and (e) bias arising from selection of reported results. Each domain was graded based on the risk level as ‘low', ‘some concerns', or ‘high' *(Supplementary file C)*.

## 3 Results

### 3.1 Study selection and characteristics

The literature search yielded a total of 2104 citations from all sources combined. During the first-level screening, 197 duplicate citations were detected and manually removed. The title-abstracts of the remaining 1907 citations were evaluated based on the PICOS criteria listed under “Eligibility criteria" (Section 2.2). Subsequently, 569 citations were deemed relevant and advanced to the second-level screening. Finally, 84 publications (of nine unique trials) satisfied the inclusion-exclusion criteria and were selected for data extraction [[35], [36], [37], [38], [39], [40], [41], [42], [43]]. All included studies were RCTs, conducted as parallel-group studies and were published between 2015 and 2023.

**Fig. 1:**
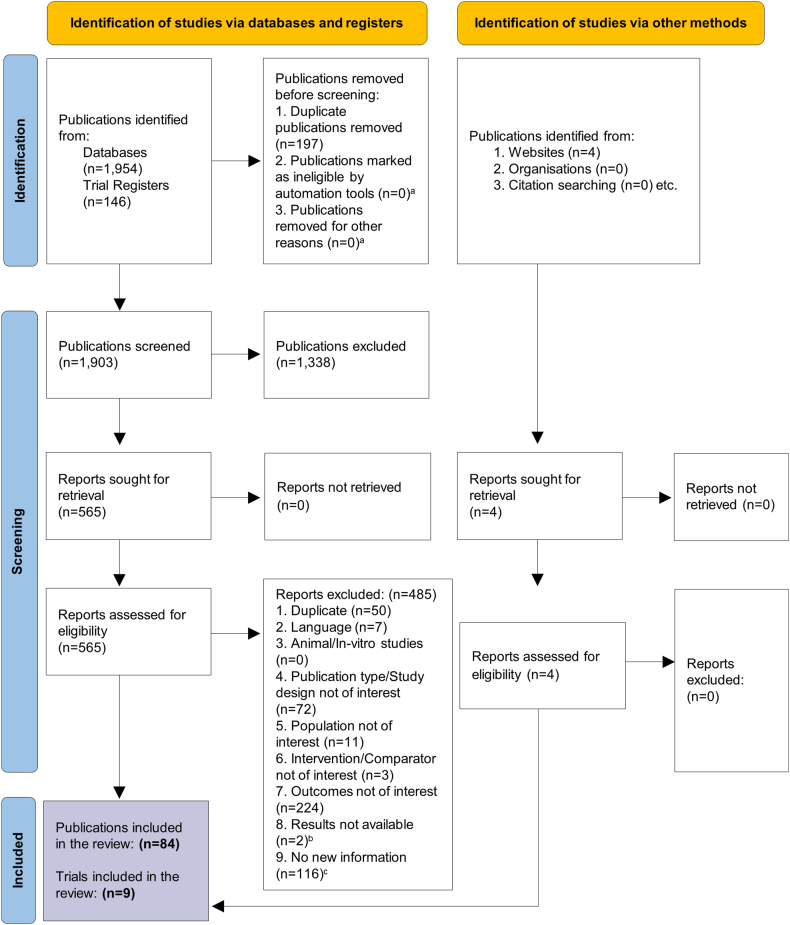
PRISMA flow diagram illustrating the study selection process ^a^Exclusion reasons listed under the “Identification" phase of PRISMA diagram are pre-listed criteria [PRISMA 2020 statement]. ^b^Reports were excluded if the study results were not reported in the full-text. ^c^Reports were excluded if they did not provide any new insights or data beyond what was already available in included primary publications. From: Page MJ, McKenzie JE, Bossuyt PM, Boutron I, Hoffmann TC, Mulrow CD et al. The PRISMA 2020 statement: an updated guideline for reporting systematic reviews. BMJ 2021; 372:n71. https://doi.org/10.1136/bmj.n71. PRISMA: Preferred reporting items for systematic reviews and meta-analyses.

Fig. 1 illustrates the PRISMA flow diagram summarising the study selection and inclusion process. Detailed characteristics of all the eligible trials and participants are reported in Table 2, Table 3, respectively. Of the nine trials included in the review, one trial compared bempedoic acid with placebo [35], one trial compared alirocumab with placebo [36], three trials compared evolocumab with different comparators [37,40,42], and four trials compared ezetimibe combined with a statin to statin monotherapy [38,39,41,43]. No published literature documenting the cardiovascular outcomes of inclisiran was identified.

**Table 2:** Characteristics of included randomised controlled trials.

Trial	Patients, N (randomised)	Trial ID	Study type	Follow-up duration	Intervention (dosage): n	Comparator (dosage): n	Primary efficacy endpoint	Key secondary efficacy endpoints	Risk of bias
**CLEAR OUTCOMES, 2023** [35]	13,970	NCT02993406	Randomised, multicentre, double-blind, Phase 3 trial	Median: 40.6 months	Bempedoic acid (180 mg/day): 6992	Placebo (dose matched to bempedoic acid): 6978	**4-component MACE**^a^	3-component MACE^b^, fatal or non-fatal MI, coronary revascularisation, fatal or non-fatal stroke, death from CV causes, death from any cause.	Low
**ODYSSEY OUTCOMES, 2018** [36]	18,924	NCT01663402	Randomised, multicentre, double-blind, Phase 3 trial	Median: 2.8 years	Alirocumab (75 mg/every 2 weeks): 9462	Placebo (dose matched to Alirocumab): 9462	**4-component MACE**^a^	4-component MACE^c^, major CHD event, any CV event, 3-component MACE^d^, death from CHD, death from CV causes, death from any cause	Low
**FOURIER, 2017** [37]	27,564	NCT01764633	Randomised, multicentre, double-blind, Phase 3 trial	Median: 2.2 years	Evolocumab (140 mg/every 2 weeks or 420 mg/every month): 13,784	Placebo (dose matched to Evolocumab): 13,780	**5-component MACE**^a^	Composite of CV death, MI, or stroke	Low
**IMPROVE-IT, 2015** [38]	18,144	NCT00202878	Randomised, multicentre, double-blind, Phase 3 trial	Median: 6 years	EZE + Statin (EZE 10 mg + Simvastatin 40 mg) once daily: 9067	Statin monotherapy (Simvastatin 40 mg + placebo) once daily: 9077	**5-component MACE**^a^	5-component MACE^e^, 5-component MACE^f^, 3-component MACE^g^	Low
**HIJ-PROPER, 2017** [39]	1734	UMIN000002742 (Japanese registry)	Randomised, multicentre, open-label, blinded-endpoint trial	3.86 years	EZE + Statin therapy (EZE 10 mg/day + Pitavastatin 2 mg/day initial dose, then adjusted to 1–4 mg/day): 864	Statin monotherapy (Pitavastatin 2 mg/day initial dose, then adjusted to 1–4 mg/day): 857	**5-component MACE**^a^	CV event (non-fatal MI, non-fatal stroke, UA, ischaemia-driven revascularisation with either PCI or CABG), all-cause death, heart failure, inflammatory markers, adverse events	Low
**Sabatine, 2015** [40]	4465	OSLER-1: NCT01439880, OSLER-2: NCT01854918	Randomised, multicentre, open-label, OSLER-1: Phase 2, OSLER-2: Phase 3	11.1 months	Evolocumab (420 mg/month in OSLER-1; 140 mg/every 2 weeks or 420 mg/month in OSLER-2): 2976	Standard therapy (based on local guidelines for the treatment of LDL-C): 1489	**3-component MACE**^a^*(Exploratory outcome*^h^*)*	Percent change in the LDL-C level. Other efficacy lipid measurements included non–HDL-C, total cholesterol, triglycerides, HDL-C, apolipoproteins A1 and B, and lipoprotein(a)	High
**RACING, 2022** [41]	3780	NCT03044665	Randomised, multicentre, open-label, Phase 4 trial	3 years	Statin + EZE (Rosuvastatin 10 mg/day + EZE 10 mg/day): 1894	Statin monotherapy (Rosuvastatin 20 mg/day): 1886	**4-component MACE**^a^	Composite of all-cause death, major CV event (coronary or peripheral revascularisation or hospitalisation for CV events) or non-fatal stroke	High
**Hao, 2022** [42]	136	NR	Prospective, Randomised	3 months	Evolocumab + atorvastatin + EZE (Evolocumab 140 mg/every 2 weeks, atorvastatin 40 mg/day, EZE 10 mg/day): 68	Atorvastatin + EZE (atorvastatin 40 mg/day and EZE 10 mg/day): 68	**4-component MACE**^a^	Adverse events	High
**Japaridze, 2017** [43]	292	NR	Randomised, single-centre, open-label	16 weeks	EZE + Atorvastatin (EZE 10 mg/day + atorvastatin 20 mg): 146	Atorvastatin monotherapy (40 mg): 146	**5-component MACE**^a^	No secondary endpoints	High

CABG: Coronary artery bypass grafting; CHD: Coronary heart disease; CV: Cardiovascular; EZE: Ezetimibe; HDL-C: High-density lipoprotein cholesterol; LDL-C: Low-density lipoprotein cholesterol; MACE: Major adverse cardiovascular events; MI: Myocardial infarction; NR: Not reported; PCI: Percutaneous coronary intervention; SC: Subcutaneous; UA: Unstable angina.

a The MACE endpoint is defined in Table 4.

b Composite of death from CV causes, non-fatal MI, or non-fatal stroke.

c Composite of death from CHD, non-fatal MI, UA requiring hospitalisation, and an ischaemia-driven coronary revascularisation.

d Composite of death from any cause, non-fatal MI, or non-fatal ischaemic stroke.

e Composite of death from any cause, major coronary event (MI, hospitalisation for UA, and coronary revascularisation), or non-fatal stroke.

f Composite CV death, non-fatal MI, hospitalisation for UA, all revascularisation, non-fatal stroke.

g Composite of CHD death, non-fatal MI, or urgent coronary revascularisation ≥30 days after randomisation.

h The primary outcome of this study was the incidence of adverse events.

**Table 3:** Baseline patient characteristics of included randomised controlled trials.

Trial	Primary vs Secondary prevention	Intervention/Comparator	Mean age in years (SD)	Female n (%)	Male n (%)	White n (%)	Region n (%)	Diabetes n (%)	Ezetimibe n (%)	ASCVD population n (%)	Statin use n (%)	Baseline LDL-C level
**CLEAR OUTCOMES, 2023** [35]	Primary prevention: 30 % Secondary prevention: 70 %	Bempedoic Acid (N = 6992)	65.5 (9.0)	3361 (48.1)	3631 (51.9) (calculated)	6397 (91.5)	NR	3144 (45.0)	803 (11.5)	CAD: 3574 (51.1) PAD: 794 (11.4) Cerebrovascular atherosclerotic disease: 1027 (14.7)	Very-low intensity: 1601 (22.9)	Mean (SD): 139.0 (34.9) mg/dL
		Placebo (N = 6978)	65.5 (8.9)	3379 (48.4)	3599 (51.6) (calculated)	6335 (90.8)	NR	3229 (46.3)	809 (11.6)	CAD: 3536 (50.7) PAD: 830 (11.9) Cerebrovascular atherosclerotic disease: 1040 (14.9)	Very-low intensity: 1573 (22.5)	Mean (SD): 139.0 (35.2) mg/dL
**ODYSSEY OUTCOMES, 2018** [36]	Secondary prevention: 100 % (Patients had previous ACS)	Alirocumab (N = 9462)	58.5 (9.3)	2390 (25.3)	7072 (74.7) (calculated)	7500 (79.3)	Central and Eastern Europe: 2719 (28.7) Western Europe: 2084 (22.0) Canada or United States: 1435 (15.2) Latin America: 1293 (13.7) Asia: 1150 (12.2) Rest of world: 781 (8.3)	2693 (28.5)	269 (2.8)	ST-segment elevation MI: 3301 (34.9) Non−ST-segment elevation MI: 4574 (48.3) Unstable angina: 1568 (16.6) Missing data: 19 (<0.1)	High-intensity: 8380 (88.6) Low/moderate-intensity: 830 (8.8) No statin: 227 (2.4)	Mean (SD): 92 (31) mg/dL Median (IQR): 87 (73–104) mg/dL
		Placebo (N = 9462)	58.6 (9.4)	2372 (25.1)	7090 (74.9) (calculated)	7524 (79.5)	Central and Eastern Europe: 2718 (28.7) Western Europe: 2091 (22.1) Canada or United States: 1436 (15.2) Latin America: 1295 (13.7) Asia: 1143 (12.1) Rest of world: 779 (8.2)	2751 (29.1)	285 (3.0)	ST-segment elevation MI 3235 (34.2) Non−ST-segment elevation MI: 4601 (48.6) Unstable angina: 1614 (17.1) Missing data: 12 (<0.1)	High-intensity: 8431 (89.1) Low/moderate-intensity: 777 (8.2) No statin: 233 (2.5)	Mean (SD): 92 (31) mg/dL Median (IQR): 87 (73–104) mg/dL
**FOURIER, 2017** [37]	Secondary prevention: 100 % (ASCVD patients)	Evolocumab (N = 13,784)	62.5 (9.1)	3387 (24.6) (calculated)	10,397 (75.4)	11,748 (85.2)	Europe: 8666 (62.9) North America: 2287 (16.6) Latin America: 913 (6.6) Asia Pacific and South Africa: 1918 (13.9)	5054 (36.7)	726 (5.3)	MI: 11,145 (80.9) Non-haemorrhagic stroke: 2686 (19.5) PAD: 1858 (13.5)	High-intensity: 9585 (69.5) Moderate-intensity: 4161 (30.2) Low-intensity, unknown intensity, or no data: 38 (0.3)	Median (IQR): 92 (80–109) mg/dL
		Placebo (N = 13,780)	62.5 (8.9)	3382 (24.5) (calculated)	10,398 (75.5)	11,710 (85.0)	Europe: 8669 (62.9) North America: 2284 (16.6) Latin America: 910 (6.6) Asia Pacific and South Africa: 1917 (13.9)	5027 (36.5)	714 (5.2)	MI: 11,206 (81.3) Non-haemorrhagic stroke: 2651 (19.2) PAD: 1784 (12.9)	High-intensity: 9518 (69.1) Moderate-intensity: 4231 (30.7) Low-intensity, unknown intensity, or no data: 31 (0.2)	Median (IQR): 92 (80–109) mg/dL
**IMPROVE-IT, 2015** [38]	Secondary prevention: 100 % (Patients had previous ACS)	Simvastatin Monotherapy (N = 9077)	63.6 (9.8)	2191 (24.1) (calculated)	6886 (75.9)	7624 (84.0)	Western Europe: 3641 (40.1) Eastern Europe: 707 (7.8) North America: 3487 (38.4) Asia Pacific: 448 (4.9) South America: 794 (8.7)	2474 (27.3)	NR	ST-segment elevation MI: 2606 (28.7) Non−ST-segment elevation MI: 4253 (46.9) Unstable angina: 2211 (24.4)	3111 (34.3)	Mean (SD): 93.8 (NR) mg/dL Median (IQR): 95.0 (79.0–110.2) mg/dL^a^
		Simvastatin + EZE (N = 9067)	63.6 (9.7)	2225 (24.5) (calculated)	6842 (75.5)	7578 (83.6)	Western Europe: 3633 (40.1) Eastern Europe: 709 (7.8) North America: 3486 (38.4) Asia Pacific: 448 (4.9) South America: 791 (8.7)	2459 (27.1)	NR	ST-segment elevation MI: 2584 (28.5) Non−ST-segment elevation MI: 4302 (47.5) Unstable angina: 2175 (24.0)	3135 (34.6)	Mean (SD): 93.8 (NR) mg/dL Median (IQR): 95.0 (79.0–110.0) mg/dL^a^
**HIJ-PROPER, 2017** [39]	Secondary prevention: 100 %	Pitavastatin monotherapy (N = 857)	65.5 (11.9)	196 (22.9) (calculated)	661 (77.1)	NR	Japan: 857 (100)	260 (30.3)	7 (0.8)	Stable angina: 100 (11.7) Previous MI: 68 (7.9) PCI: 75 (8.8) CABG: 8 (0.9) Chronic heart failure: 15 (1.8) Cerebrovascular disease: 49 (5.7) PAD: 17 (2.0)	149 (17.4)	Mean (SD): 135.6 (30.0) mg/dL
		Pitavastatin + EZE (N = 864)	65.7 (11.7)	225 (26.0) (calculated)	639 (74.0)	NR	Japan: 864 (100)	260 (30.1)	12 (1.4)	Stable angina: 98 (11.3) Previous MI: 62 (7.2) PCI: 71 (8.2) CABG: 10 (1.2) Chronic heart failure: 21 (2.4) Cerebrovascular disease: 56 (6.5) PAD: 15 (1.7)	143 (16.6)	Mean (SD): 134.8 (29.3) mg/dL
**Sabatine, 2015** [40]	NR	Evolocumab (N = 2976)	57.8 (11.0)	1486 (49.9) (calculated)	1490 (50.1)	2559 (86.0)	Europe: 1205 (40.5) North America: 1402 (47.1) Asia Pacific or South Africa: 369 (12.4)	382 (12.8)	376 (12.6)	MI: 276 (9.3) PCI: 325 (10.9) CABG: 185 (6.2) Carotid- or vertebral-artery disease: 94 (3.2) Stroke: 81 (2.7) PAD: 85 (2.9)	Any statin: 2073 (69.7) High-intensity: 795 (26.7) Moderate-intensity: 1034 (34.7) Low-intensity: 240 (8.1)	Median (IQR): 120 (97–148) mg/dL
		Standard therapy (N = 1489)	58.2 (10.9)	724 (48.6) (calculated)	765 (51.4)	1267 (85.1)	Europe: 597 (40.1) North America: 705 (47.3) Asia Pacific or South Africa: 187 (12.6)	217 (14.6)	229 (15.4)	MI: 141 (9.5) PCI: 170 (11.4) CABG: 110 (7.4) Carotid- or vertebral-artery disease: 62 (4.2) Stroke: 37 (2.5) PAD: 50 (3.4)	Any Statin: 1055 (70.9) High-intensity: 415 (27.9) Moderate-intensity: 522 (35.1) Low-intensity: 118 (7.9)	Median (IQR): 121 (97–151) mg/dL
**RACING, 2022** [41]	Secondary prevention: 100 %	Moderate-intensity statin with EZE combination therapy (N = 1894)	64 (10)	474 (25)	1420 (75)	NR	South Korea: 1894 (100)	701 (37)	With high-intensity statin: 85 (4) With moderate-intensity statin: 251 (13)	Previous MI: 744 (39) Previous PCI: 1258 (66) Previous CABG: 132 (7) ACS: 27 (1) Previous Ischaemic stroke: 101 (5) PAD: 66 (4)	High-intensity: 711 (38) High-intensity with EZE: 85 (4) Moderate-intensity: 681 (36) Moderate-intensity with EZE: 251 (13) Low-intensity: 6 (<1) None: 160 (8)	Median (IQR): 80 (64–100) mg/dL
		High-intensity statin monotherapy (N = 1886)	64 (10)	480 (25)	1406 (75)	NR	South Korea: 1886 (100)	697 (37)	With high-intensity statin: 63 (3) With moderate-intensity statin: 248 (13)	Previous MI: 745 (40) Previous PCI: 1239 (66) Previous CABG: 115 (6) ACS: 20 (1) Previous Ischaemic stroke: 112 (6) PAD: 69 (4)	High-intensity: 729 (39) High-intensity with EZE: 63 (3) Moderate-intensity: 685 (36) Moderate-intensity with EZE: 248 (13) Low-intensity: 5 (<1) None: 156 (8)	Median (IQR): 80 (64–100) mg/dL
**Hao, 2022** [42]	Secondary prevention: 100 %	Evolocumab + EZE + Atorvastatin (N = 68)	62.21 (12.31)	23 (33.82) (calculated)	45 (66.18)	NR	NR	27 (39.71)	NR	ST-segment elevation MI: 27 (39.7) Non−ST-segment elevation MI: 34 (50.0) Unstable angina: 7 (10.3)	NR	Mean (SD): 3.54 (0.58) mmol/L
		EZE + Atorvastatin (N = 68)	62.22 (11.44)	20 (29.41) (calculated)	48 (70.59)	NR	NR	23 (33.82)	NR	ST-segment elevation MI: 28 (41.2) Non−ST-segment elevation MI: 31 (45.6) Unstable angina: 9 (13.2)	NR	Mean (SD): 3.52 (0.41) mmol/L
**Japaridze, 2017** [43]	Secondary prevention: 100 % (Patients with ACS)	EZE + Atorvastatin (N = 146)	62.21 (11.36)	67 (45.9)	79 (54.1) (calculated)	NR	Georgia: 146 (100)	7 (4.8)	0 (0)	PAD: 58 (39.7) Old MI: 25 (17.1) CABG: 23 (15.8) PCI: 12 (8.2) Stroke or TIA: 21 (14.4)	NR	Mean (SD): 2.83 (0.55) mmol/L
		Atorvastatin (N = 146)	62.62 (11.03)	68 (46.9)	78 (54.1) (calculated)	NR	Georgia: 146 (100)	2 (1.4)	0 (0)	PAD: 67 (45.9) Old MI: 4 (2.7) CABG: 12 (8.2) PCI: 22 (15.1) Stroke or TIA: 39 (26.7)	NR	Mean (SD): 2.74 (0.64) mmol/L

ACS: Acute coronary syndrome; ASCVD: Atherosclerotic cardiovascular disease; CABG: Coronary artery bypass grafting; CAD: Coronary artery disease; CHD: Coronary heart disease; CHF: Congestive heart failure; CKD: Chronic kidney disease; CV: Cardiovascular; EZE: Ezetimibe; IQR: Interquartile range; LDL-C: Low-density lipoprotein cholesterol; MI: Myocardial infarction; N: Patients randomised; n: Patients analysed; NR: Not reported; PAD: Peripheral artery disease; PCI: Percutaneous coronary intervention; SD: Standard deviation; TIA: Transient ischaemic stroke.

a Baseline data were available for 8990 participants in the simvastatin–ezetimibe and for 9009 participants in the simvastatin monotherapy group.

**Table 4:** Primary MACE outcomes in included randomised controlled trials.

Trial	MACE endpoint definition	Outcome measure
**CLEAR OUTCOMES, 2023** [35]	**4-component MACE:** Composite of CV death, non-fatal MI, non-fatal stroke, or coronary revascularisation	Bempedoic acid vs Placebo HR: 0.87 (95 % CI: 0.79–0.96) (P = 0.004)
**ODYSSEY OUTCOMES, 2018** [36]	**4-component MACE:** Composite of CHD-death, non-fatal MI, fatal or non-fatal ischaemic stroke, or UA requiring hospitalisation	Alirocumab vs Placebo HR: 0.85 (95 % CI: 0.78–0.93) (P < 0.001)
**FOURIER, 2017** [37]	**5-component MACE:** Composite of CV death, MI, stroke, hospitalisation for UA, or coronary revascularisation	Evolocumab vs Placebo HR: 0.85 (95 % CI: 0.79–0.92) (P < 0.001)
**IMPROVE-IT, 2015** [38]	**5-component MACE:** Composite of death from CVD, a major coronary event (non-fatal MI, documented UA requiring hospital admission, or coronary revascularisation), or non-fatal stroke	Ezetimibe + Simvastatin vs Simvastatin HR: 0.936 (95 % CI: 0.89–0.99) (P = 0.016)
**HIJ-PROPER, 2017** [39]	**5-component MACE:** Composite of all-cause death, non-fatal MI, non-fatal stroke, UA, or revascularisation with either PCI or CABG	Ezetimibe + Pitavastatin vs Pitavastatin HR: 0.89 (95 % CI: 0.76–1.04) (P = 0.152)
**Sabatine, 2015** [40]	**3-component MACE:** Composite of death, major coronary events, or major cerebrovascular events^a^	Evolocumab vs Standard therapy HR: 0.47 (95 % CI: 0.28–0.78) (P = 0.003)
**RACING, 2022** [41]	**4-component MACE:** Occurrence of CV death, major CV events (coronary or peripheral revascularisation, hospitalisation for CV events), or non-fatal stroke	Ezetimibe + Rosuvastatin vs Rosuvastatin HR: 0.92 (95 % CI: 0.75–1.13) (P = 0.43)
**Hao, 2022** [42]	**4-component MACE:** Composite of cardiogenic death, non-fatal MI, non-fatal stroke, or readmission due to angina	Evolocumab + Ezetimibe + Atorvastatin vs Ezetimibe + Atorvastatin (8.82 % vs 24.59 %) (P = 0.015)^b^
**Japaridze, 2017** [43]	**5-component MACE:** Composite of death from CVD, non-fatal MI, UA requiring hospital admission, coronary revascularisation, or non-fatal stroke	Atorvastatin vs Ezetimibe + Atorvastatin HR: 2.099 (95 % CI: 1.165–3.781) (P = 0.014)

CABG: Coronary artery bypass grafting; CHD: Coronary heart disease; CI: Confidence interval; CV: Cardiovascular; CVD: Cardiovascular disease; HR: Hazard ratio; MACE: Major adverse cardiovascular events; MI: Myocardial infarction; PCI: Percutaneous coronary intervention; UA: Unstable angina.

a Exploratory outcome.

b Hazard ratio not reported in the trial.

### 3.2 Major adverse cardiovascular events (MACE)

The primary outcome of eight of the nine included trials was either a 3-component, 4-component, or 5-component MACE event. Out of the nine trials analysed, four (CLEAR Outcomes, ODYSSEY Outcomes, RACING, and Hao 2022) had a four-component MACE as the primary endpoint [35,36,41,42], while the other four trials (FOURIER, IMPROVE-IT, HIJ-PROPER, and Japaridze 2017) had a five-component MACE as the primary endpoint [[37], [38], [39],^43^]. Only one trial (Sabatine 2015) reported three-component MACE as an exploratory outcome [40]. However, the definition of MACE varied among the trials and is detailed in Table 4. It is important to note that not all MACE components carry equal importance or robustness, with hospitalisation for unstable angina being one of the less robust components included.

#### 3.2.1 Bempedoic acid

In the CLEAR OUTCOMES trial, treatment with bempedoic acid was associated with a lower risk of four-component MACE among statin-intolerant patients. The incidence of the primary four-component MACE endpoint (composite of cardiovascular death, non-fatal MI, non-fatal stroke, or coronary revascularisation) was significantly reduced in patients treated with bempedoic acid compared to those treated with placebo (11.7 % (819 patients) vs 13.3 % (927 patients); HR (hazard ratio): 0.87; 95 % CI: 0.79–0.96; P = 0.004) [35]. Further, the mean placebo-adjusted LDL-C reduction with bempedoic acid (in the intention-to-treat analysis) at one year was 0.58 mmol/L (95 % CI: 0.55–0.61 mmol/L) [19]. When normalised per 1 mmol/L reduction in LDL-C as per the CTTC methodology, the HR for major vascular event composite endpoint was 0.75 (95 % CI: 0.63–0.90), similar to the rate ratio of 0.78 (95 % CI: 0.76–0.80) observed for statins in the 2010 CTTC meta-analysis [18,19].

#### 3.2.2 Alirocumab

In the ODYSSEY OUTCOMES trial, the primary four-component MACE endpoint event (composite of coronary heart disease-death, non-fatal MI, fatal or non-fatal ischaemic stroke, or hospitalisation for unstable angina) occurred in 9.5 % (903) patients in the alirocumab group and 11.1 % (1052) patients in the placebo group (HR: 0.85; 95 % CI: 0.78–0.93; P < 0.001) [36]. Further, at one year, the mean LDL-C level in the alirocumab group was lower than in the placebo group (1.2 mmol/L vs 2.5 mmol/L, respectively) [36].

#### 3.2.3 Evolocumab

In the FOURIER trial, evolocumab significantly reduced the risk of the primary five-component MACE endpoint (composite of cardiovascular death, MI, stroke, hospitalisation for unstable angina, or coronary revascularisation) [37]. The primary endpoint occurred in 9.8 % (1344) of patients treated with evolocumab, compared to 11.3 % (1563) of patients in the placebo group, with a hazard ratio of 0.85 (95 % CI: 0.79–0.92; P < 0.001). At 48 weeks, the mean absolute reduction in LDL-C levels with evolocumab, compared to placebo, was 1.45 mmol/L [95 % CI, 1.43 to 1.47]) [37].

In an analysis of the OSLER-1 and 2 trials, a reduced risk of three-component MACE endpoint (composite of death, major coronary events, or major cerebrovascular events) (HR: 0.47; 95 % CI: 0.28–0.78; P = 0.003) was reported in the evolocumab group compared to standard therapy [40].

In Hao 2022, the incidence of four-component MACE endpoint (composite of cardiogenic death, non-fatal MI, non-fatal stroke, or readmission due to angina) was lower in the evolocumab group than in the control group (8.82 % vs 24.59 %, P = 0.015) [42].

#### 3.2.4 Ezetimibe

In the IMPROVE-IT trial, the primary five-component MACE endpoint (composite of death from CVD, non-fatal MI, documented hospitalisation for unstable angina, coronary revascularisation, or non-fatal stroke) occurred in 32.7 % (2572) patients in the simvastatin plus ezetimibe group and 34.7 % (2742) patients in the simvastatin monotherapy group (HR: 0.936; 95 % CI: 0.89–0.99; P = 0.016) [38]. At one year, the mean LDL-C level in the simvastatin–ezetimibe group was 0.43 mmol/L lower than in the simvastatin-monotherapy group (1.4 mmol/L vs 1.8 mmol/L, P < 0.001) [38]. The HR for clinical benefit per millimole of LDL-C reduction with ezetimibe was 0.80, compared to 0.78 for statins as reported in the CTTC meta-analysis [18].

In the HIJ-PROPER trial, the incidence of the primary five-component MACE endpoint (composite of all-cause death, non-fatal MI, non-fatal stroke, unstable angina, or revascularisation with either percutaneous coronary intervention or coronary artery bypass grafting) was 36.9 % with pitavastatin monotherapy and 32.8 % with pitavastatin plus ezetimibe [39]. However, this difference was not statistically significant (HR: 0.89; 95 % CI: 0.76–1.04; P = 0.152).

In the RACING trial, the primary four-component MACE endpoint (occurrence of cardiovascular death, coronary or peripheral revascularisation, hospitalisation for cardiovascular events, or non-fatal stroke) occurred in 9.1 % (172) patients in the moderate-intensity statin plus ezetimibe group and 9.9 % (186) patients in the high-intensity statin monotherapy group (HR: 0.92; 95 % CI: 0.75–1.13; P = 0.43) [41].

In Japaridze 2017, the combination therapy of atorvastatin and ezetimibe was associated with a significantly reduced risk of cardiovascular events compared to statin monotherapy, with an 11.1 % lower rate (HR: 2.099; 95 % CI: 1.165–3.781; P = 0.014) of the primary five-component MACE endpoint (composite of death from CVD, non-fatal MI, hospitalisation for unstable angina, coronary revascularisation, or non-fatal stroke) [43].

#### 3.2.5 Inclisiran

At the time of conduct of this SLR, no published literature documenting the cardiovascular outcomes of inclisiran was identified. However, three trial records were identified through trial registry searches: ORION-4 (NCT03705234) and VICTORIAN-2 PREVENT (NCT05030428) trials for secondary prevention patients, and VICTORIAN-1 PREVENT (NCT05739383) trial for primary prevention patients [[44], [45], [46]].

### 3.3 Safety

In the CLEAR Outcomes trial, the overall incidence rate of AEs, serious AEs, and AEs leading to treatment discontinuation did not differ meaningfully between the bempedoic acid group and the placebo group [35]. There were slight increases in serum creatinine and uric acid levels with bempedoic acid compared to placebo [35,47]. These changes were probably related to inhibition of the renal transporter – organic anion transporter 2 (OAT2) and reflect small changes in renal laboratories rather than clinically meaningful changes in renal function [47,48].

The incidence of AEs was comparable between the alirocumab group and the placebo group, except for local injection-site reactions being more common with alirocumab than placebo (3.8 % vs 2.1 %) [36]. Similarly, there was no significant difference between the evolocumab group and the placebo group with regards to overall rates of AEs, treatment-related AEs, serious AEs, or AEs leading to treatment discontinuation. However, injection-site reactions were more common with evolocumab in comparison to placebo (2.1 % vs 1.6 %) [37]. Furthermore, there were no significant differences in the safety profile and rate of treatment discontinuation due to AEs between the simvastatin plus ezetimibe group and the simvastatin monotherapy group [38].

### 3.4 Quality assessment of included trials

**Fig. 2:**
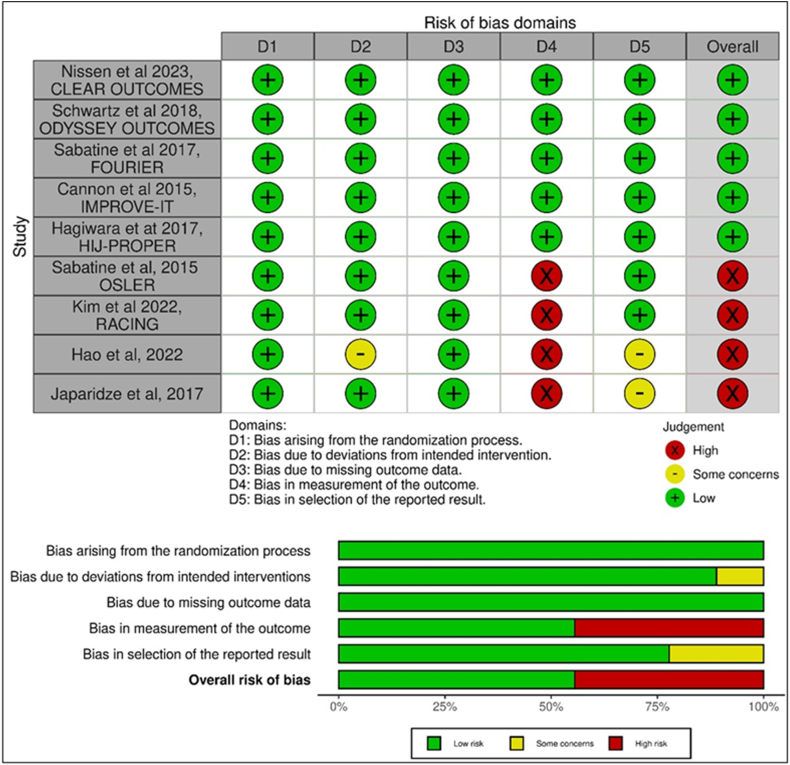
Risk of bias graph.

The Cochrane RoB 2 [34] was utilised for the quality assessment of all included trials (n = 9) (Fig. 2). Results revealed that all trials used a randomised trial design, with allocation concealment reported in only one trial [36]. Four trials had adequate double-blind settings [[35], [36], [37], [38]]; four were open-label [[39], [40], [41],^43^], and one did not report blinding information [42]. All trials demonstrated a low risk of bias in missing outcomes data, but one had concerns regarding deviations from the intended intervention [42]. Four open-label trials exhibited a high risk of bias in outcome measurement due to AEs reporting bias [[39], [40], [41],^43^], and two showed concerns of bias by not reporting data analysis according to a pre-specified plan [42,43]. Overall, the quality of included RCTs in this SLR ranged from fair to good *(Supplementary file D)*.

## 4 Discussion

### 4.1 Interpretation of results

The present SLR identified and evaluated the cardiovascular outcomes trials of non-statin LLTs – bempedoic acid, ezetimibe, alirocumab and evolocumab. Findings of this SLR suggest that non-statin LLTs play a crucial role in reducing cardiovascular risk in adults with established CVD or at high cardiovascular risk. While statins remain the primary class of LLTs used in clinical practice, other classes such as ACL inhibitors, PCSK9 inhibitors, and ezetimibe have also shown promising results in terms of their safety and efficacy [21,49].

However, it is crucial to recognise that the efficacy and safety of LLTs can vary depending on the patient population studied and individual patient characteristics. Only one identified trial (CLEAR OUTCOMES – bempedoic acid) evaluated the benefits of cardiovascular risk reduction among primary prevention and statin-intolerant population [35]. In this trial, cardiovascular risk reduction with bempedoic acid was found to be similar to that achieved with statins for a given absolute magnitude of LDL-C lowering [19]. A comprehensive understanding of bempedoic acid's mechanism, metabolism, and side effects has highlighted its potential benefits, offering a promising alternative for cardiologists and clinicians facing the challenges of managing muscle-related side effects from statins [50].

Four different trials evaluating alirocumab and evolocumab reported reduced cardiovascular events in patients who are either not suitable for alternative lipid-lowering medications or those who fail to achieve their lipid targets with conventional treatments (including statins) [36,37,40,42]. The combination therapy of ezetimibe and statins also showcased significant reduction in cardiovascular risk compared to statin monotherapy, highlighting its utility as an adjunctive treatment option [38,39,41,43]. Three trial registries were identified, documenting the ongoing cardiovascular outcomes trials involving inclisiran [[44], [45], [46]]. Since the trials are ongoing, no data on its efficacy and safety is available. Overall, our findings highlight the effectiveness of non-statin LLTs in mitigating cardiovascular risk among high/very-high risk patients. With the exception of alirocumab and evolocumab showing a higher incidence of injection-site reactions [36,37], non-statin LLTs seemed to be well tolerated and were comparable in terms of safety to the comparator treatments studied in individual trials.

### 4.2 Comparison with other studies

Many previously published SLRs and meta-analyses, including pooled analyses, have assessed the LDL-C lowering benefits of LLTs [[51], [52], [53], [54]]. However, few studies have specifically examined the cardiovascular benefits of these therapies. Previous reviews that evaluated the cardiovascular benefits of LLTs, did not include bempedoic acid, unlike our review [[55], [56], [57]]. Additionally, previous studies often included trials where MACE endpoints were analysed as safety events rather than efficacy endpoints [58,59]. Overall, our review represents the first comprehensive SLR to summarise all the prominent cardiovascular outcomes trials (CVOT) of non-statin LLTs – bempedoic acid, alirocumab, ezetimibe, and evolocumab.

### 4.3 Implications of the findings

Non-statin therapies are often combined with statins to consistently and progressively reduce LDL-C levels (especially in very high risk patients). This reduction is crucial for achieving and sustaining cardiovascular benefits [60]. The implications of our findings emphasise the significance of considering non-statin LLTs as viable treatment options for individuals with CVD or high cardiovascular risk who cannot tolerate or achieve optimal lipid control with statin therapy alone. Bempedoic acid and ezetimibe have also shown to reduce high-sensitivity C-reactive protein (hs-CRP) levels, a key inflammation biomarker, independent of their LDL-C lowering effects. This suggests that these lipid-lowering therapies not only contribute to cardiovascular risk reduction by lowering cholesterol but also exert additional anti-inflammatory effects, which may further enhance their overall cardiovascular protective benefits [35,38,61]. Incorporating these alternative therapies into clinical practice guidelines may improve cardiovascular outcomes and reduce the burden of CVD-related morbidity and mortality. Our results highlight the need for personalized treatment strategies tailored to individual patient profiles, including factors such as statin intolerance, comorbidities, and treatment preferences. By offering a diverse array of LLTs, clinicians can optimise cardiovascular risk management and increase patient adherence.

### 4.4 Strengths and limitations

A notable strength of this systematic review is its comprehensive analysis of multiple non-statin LLTs, providing a holistic understanding of their cardiovascular efficacy and safety. Moreover, our rigorous methodology, adherence to established guidelines, and thorough quality assessment enhance the reliability and validity of our findings. However, several limitations warrant consideration. Firstly, inconsistencies in MACE definitions across studies posed challenges in comparing outcomes, with variations observed in the components considered for MACE definitions. Additionally, discrepancies were noted in other baseline characteristics such as the percentage of females, regional demographics, background ezetimibe usage, baseline LDL-C levels, and the proportion of patients receiving statin treatment at baseline. Furthermore, heterogeneity was observed across studies, particularly regarding background statin usage. Variations in background statin utilisation were evident, with the CLEAR Outcomes study primarily focusing on a population intolerant to statins [35]. In contrast, other studies included individuals with mixed levels of statin usage at baseline. Including only English-language publications may also introduce language bias, potentially overlooking relevant studies published in other languages.

While the studies included in this SLR focused on CV risk reduction over their respective trial periods, it is recognised that earlier reductions in LDL-C are beneficial, as they reduce the time for which patients are exposed to high LDL-C levels. An important step towards quantifying this benefit using data from short-term randomised trials is to accurately measure LDL-C levels at regular intervals. This would allow monitoring the reduction in major cardiovascular events per plaque–year (mmol/l) reduction in cumulative exposure to LDL at each time point [62].

## 5 Conclusion

In summary, our study summarises and highlights the efficacy and safety of non-statin LLTs, including bempedoic acid, alirocumab, evolocumab, ezetimibe, and inclisiran, in reducing cardiovascular risk among adults with established CVD or at high cardiovascular risk. These findings support the integration of non-statin LLTs into clinical practice guidelines as adjunctive therapies for individuals unable to achieve optimal lipid control with statins alone. Future research should focus on elucidating the long-term cardiovascular benefits and safety profiles of these therapies, particularly in diverse patient populations, to further inform evidence-based treatment strategies for CVD prevention and management. It is important to acknowledge that there was considerable variation in the patient populations and the outcomes reported across the trials. Future research may consider conducting an indirect treatment comparison/meta-analysis while considering the heterogeneity across trials.

## Financial sources

This work was supported by 10.13039/501100022274Daiichi Sankyo Europe GmbH and conducted by Skyward Analytics Pvt. Ltd.

## Declaration of competing interest

The authors declare the following financial interests/personal relationships which may be considered as potential competing interests: Prof. Gabriella Iannuzzo reports a relationship with SOBI, Daiichi-Sankyo, Novartis that includes: participation in advisory board. Prof. Gabriella Iannuzzo reports a relationship with 10.13039/100004339Sanofi, 10.13039/100002429Amgen, 10.13039/100013220Ultragenyx that includes: support for attending meetings. Prof. Gabriella Iannuzzo reports a relationship with Ultragenyx and Amryt Pharmaceuticals that includes: internal training. Shantanu Jawla reports a relationship with Daiichi-Sankyo that includes: employment. Geetank Kamboj reports a relationship with Skyward Analytics that includes: employment. Geetank Kamboj reports a relationship with Daiichi-Sankyo that includes: consulting or advisory. Parinita Barman reports a relationship with Skyward Analytics that includes: employment. Parinita Barman reports a relationship with Daiichi-Sankyo that includes: consulting or advisory. Shirish Dongare reports a relationship with Skyward Analytics that includes: employment. Shirish Dongare reports a relationship with Daiichi-Sankyo that includes: consulting or advisory. There are no other known competing financial interests or personal relationships that could have influenced the work reported in this paper.

## Abbreviations

ACC: American College of Cardiology
ACL: Adenosine triphosphate-citrate lyase inhibitors
AEs: Adverse events
AHA: American Heart Association
ASCVD: Atherosclerotic cardiovascular disease
CTTC: Cholesterol Treatment Trialists' Collaboration
CVD: Cardiovascular diseases
CVOT: Cardiovascular outcomes trial
EAS: European Atherosclerosis Society
ESC: European Society of Cardiology
HR: Hazard ratio
hs-CRP: High-sensitivity C-reactive protein
ICTRP: International Clinical Trials Registry Platform
LDL-C: Low-density lipoprotein cholesterol
LLTs: Lipid-lowering therapies
MACE: Major adverse cardiovascular events
MI: Myocardial infarction
OAT2: Organic Anion Transporter 2
PCSK9: Proprotein convertase subtilisin/kexin type 9 inhibitors
PICOS: Population, intervention, comparators, outcome(s), and study design
PRISMA: Preferred Reporting Items for Systematic Reviews and Meta-Analyses
RCTs: Randomised controlled trials
RoB 2: Cochrane Risk-of-Bias 2 tool
SLR: Systematic literature review
WHO: World Health Organisation
