# Supplementary material for: Efficacy and safety of lipid-lowering therapies in combination with or without statin to reduce the cardiovascular risk: A systematic review of randomised controlled trials
Source: Atheroscler Plus. 2024 Oct 17;58:24–37. doi: 10.1016/j.athplu.2024.10.001 (PMC11541451; doi:10.1016/j.athplu.2024.10.001)
Supplement: Multimedia component 2 [file mmc2.docx]

Database search terms for systematic review
Embase and Medline (via OVID)
Cardiovascular disease related terms
'cardiovascular disease':ab,ti,de OR 'cardio*':ab,ti,de OR 'cardia*':ab,ti,de OR 'heart*':ab,ti,de OR 'coronary*':ab,ti,de OR 'coronary risk factor*':ab,ti,de OR 'myocard*':ab,ti,de OR 'isch?em*':ab,ti,de OR 'emboli*':ab,ti,de OR 'thrombo*':ab,ti,de OR 'ventric*':ab,ti,de OR 'arrhythmi*':ab,ti,de OR 'atrial fibrillat*':ab,ti,de OR 'tachycardi*':ab,ti,de OR stroke:ab,ti,de OR strokes:ab,ti,de OR 'cerebrovascular disease':ab,ti,de OR 'arteriosclerosis':ab,ti,de
Interventions/Comparators related terms
'bempedoic acid'/exp OR 'bempedoic acid':ab,ti,de OR nilemdo:ab,ti,de OR nexletol:ab,ti,de OR 'etc 1002':ab,ti,de OR esp55016:ab,ti,de OR 'esp 55016':ab,ti,de OR 'alirocumab'/exp OR 'alirocumab':ab,ti,de OR 'praluent':ab,ti,de OR 'regn 727':ab,ti,de OR 'regn727':ab,ti,de OR 'sar 236553':ab,ti,de OR 'sar236553':ab,ti,de OR 'evolocumab'/exp OR 'evolocumab':ab,ti,de OR 'amg 145':ab,ti,de OR 'amg145':ab,ti,de OR 'repatha':ab,ti,de OR 'ezetimibe'/exp OR 'ezetimibe':ab,ti,de OR 'ezetib':ab,ti,de OR 'ezetimib':ab,ti,de OR 'absorcol':ab,ti,de OR 'ezetrol':ab,ti,de OR 'sch 58235':ab,ti,de OR 'sch58235':ab,ti,de OR 'viemm':ab,ti,de OR 'zetia':ab,ti,de OR 'zient':ab,ti,de OR nustendi:ab,ti,de OR nexlizet:ab,ti,de OR inclisiran:ab,ti,de OR 'aln-pcssc':ab,ti,de OR leqvio:ab,ti,de
Study design related terms
((cross NEXT/1 over*):ab,ti,de) OR 'placebo* next/1 control*':ab,ti,de OR ((doubl* NEAR/1 blind*):ab,ti,de) OR ((singl* NEAR/1 blind*):ab,ti,de) OR 'randomi?ed controlled trial$':ab,ti,de OR ((random$ NEXT/2 allocat$):ab,ti,de) OR 'phase 3 clinical trial':ab,ti,de OR 'phase 2 clinical trial':ab,ti,de OR 'controlled clinical trial':ab,ti,de OR 'systematic next/2 review':ab,ti,de OR 'systematic review'/de OR 'meta-analysis'/de OR 'meta analysis'/de OR 'network meta-analysis'/de
Exclusion terms
('animal'/exp NOT 'human'/exp) OR comment*:ti OR 'letter':ti OR [letter]/lim OR 'editorial':ti OR [editorial]/lim   OR 'case stud*':ti OR 'case report':ti OR 'case series':ti OR 'narrative review*':ti OR [review]/lim OR 'in-vitro':ti OR 'in vitro':ti OR [erratum]/lim

Cochrane Library
Cardiovascular disease related terms
MeSH descriptor: [Cardiovascular Diseases] this term only OR 'cardio*':ab,ti OR 'cardia*':ab,ti OR 'heart*':ab,ti OR 'coronary*':ab,ti OR 'myocard*':ab,ti OR 'isch?em*':ab,ti OR 'emboli*':ab,ti OR 'ventric*':ab,ti OR 'arrhythmi*':ab,ti OR 'thrombo*':ab,ti OR 'atrial fibrillat*':ab,ti OR 'tachycardi*':ab,ti OR 'coronary risk factor*':ab,ti OR stroke:ab,ti OR strokes:ab,ti OR MeSH descriptor: [Cerebrovascular Disorders] this term only OR MeSH descriptor: [Arteriosclerosis] this term only
Interventions/Comparators related terms
'Bempedoic acid':ab,ti OR nilemdo:ab,ti OR nexletol:ab,ti OR 'ETC-1002' OR ESP55016:ab,ti OR 'ESP-55016':ab,ti OR ezetimibe:ab,ti OR ezetrol:ab,ti OR zetia:ab,ti OR sch58235:ab,ti OR 'sch-58235':ab,ti OR 'sch 58235':ab,ti OR nustendi:ab,ti OR nexlizet:ab,ti OR alirocumab:ab,ti OR sar236553:ab,ti OR 'sar 236553':ab,ti OR 'regn 727':ab,ti OR 'monoclonal antibody regn727':ab,ti OR 'regn727 monoclonal antibody':ab,ti OR regn727:ab,ti OR praluent:ab,ti OR evolocumab:ab,ti OR repatha:ab,ti OR 'amg-145':ab,ti OR 'amg 145':ab,ti OR inclisiran:ab,ti OR 'aln-pcssc':ab,ti OR leqvio:ab,ti
